# Low-Grade Primary Splenic CD10-Positive Small B-Cell Lymphoma/Follicular Lymphoma

**DOI:** 10.3390/curroncol28060407

**Published:** 2021-11-18

**Authors:** Rami Abdulbaki, Parastou Tizro, Victor E. Nava, Maria Gomes da Silva, João L. Ascensão

**Affiliations:** 1 Department of Pathology, George Washington University, Washington, DC 20037, USA; Rabdulbaki@gwu.edu (R.A.); victor.nava@va.gov (V.E.N.); 2 City of Hope Medical Canter, Department of Pathology, Duarte, CA 91010, USA; ptizro@coh.org; 3 Veterans Affairs Medical Center, Washington, DC 20052, USA; 4 Department of Hematology, Initituto Português de Oncologia, 1649-028 Lisboa, Portugal; mgsilva@ipolisboa.min-saude.pt; 5 Veterans Affairs Medical Center, Department of Hematology, Washington, DC 20052, USA

**Keywords:** low-grade B-cell lymphomas, follicular lymphoma, primary splenic follicular lymphoma

## Abstract

Primary splenic lymphoma (PSL) is a rare malignancy representing about 1% of all lymphoproliferative disorders, when using a strict definition that allows only involvement of spleen and hilar lymph nodes. In contrast, secondary low-grade B-cell lymphomas in the spleen, such as follicular lymphomas (FL), lymphoplasmacytic lymphoma and chronic lymphocytic leukemia/ small lymphocytic lymphoma, particularly as part of advanced stage disease, are more common. Indolent B cell lymphomas expressing CD10 almost always represent FL, which in its primary splenic form is the focus of this review. Primary splenic follicular lymphoma (PSFL) is exceedingly infrequent. This type of lymphoproliferative disorder is understudied and, in most cases, clinically characterized by splenomegaly or cytopenias related to hypersplenism. The diagnosis requires correlation of histopathology of spleen, blood and/or bone marrow with the correct immunophenotype (determined by flow cytometry and/or immunohistochemistry) and if necessary, additional molecular profiling. Management of this incurable disease is evolving, and splenectomy remains the mainstream treatment for stage I PSFL.

## 1. Introduction

Neprilysin (CD10) is a cell surface zinc-dependent metalloendopeptidase expressed in normal and neoplastic hematopoietic and non-hematopoietic tissue [1]. This protein was first identified in kidney and found afterwards to be also expressed widely in other tissues using immunohistochemistry [2,3]. The protein expression profile of CD10, includes frequently lymphoblasts from children with acute lymphoblastic leukemia (ALL), which led to the descriptor of Common ALL Antigen (CALLA) for this enzyme. In hematopoietic tissue, CD10 is detected in lymphocytes (in a maturation dependent fashion) and neutrophils [3]. Benign and neoplastic B cells of germinal center derivation, and immature lymphoid cells are positive for CD10. Therefore, detection of this antigen (in conjunction with CD5 and CD23) is invaluable for the classification of mature and immature lymphoid neoplasms (see below) and was used (together with CD5) to organize this review series (Aggarwal, Auerbach, Gomes da Silva Cross Reference). Besides its crucial diagnostic value, the potential prognostic role of CD10 expression was recognized early [4]. Therefore, CD10 constitutes a canonical element of current subclassification and prognostic algorithms in diffuse large B cell lymphoma (DLBCL), where its expression generally predicts more favorable outcomes [5]. However, CD10 cannot be used alone for independent prognostication of low-grade B cell lymphoma.

CD10 expression together with light chain restriction (monoclonality) in a low-grade B-cell population supports the diagnosis of follicular lymphoma (FL), a very common disease accounting for 10 to 20% of all lymphomas in the Western world. However, CD10 positivity cannot be used in isolation for the diagnosis of FL, since tumor expression varies depending on the idiosyncrasy and the topography of the neoplasm. For instance, CD10 expression was present in 77% of 127 cases of FL diagnosed in a subspecialized pathology consultation service [6]. Furthermore, high-grade lymphomas/leukemias, such as DLBCL or Burkitt lymphoma (BL) are also classically positive for CD10. In addition, lymphomas that are typically negative for CD10, including marginal zone lymphoma (MZL), hairy cell leukemia (HCL) and lymphoplasmacytic lymphomas (LPL), can occasionally be reactive for this antigen [7]. Finally, very rare instances of CD5/CD10-double positive B cell lymphomas have been reported [8]. Therefore, integration of clinicopathologic data is necessary for an accurate diagnosis of lymphomas, including multiparametric marker evaluation where CD10 expression is a critical value.

Follicular lymphoma (FL) is a mature B-cell non-Hodgkin lymphoma derived from germinal center B-lymphocytes (centrocytes and centroblasts) that generally has at least a partially follicular/nodular histologic pattern [9]. Nodal disease is most common, and extension to any extranodal site may ensue [10]. Lymphomatous involvement of bone marrow, peripheral blood, and spleen (seen in 20% of cases) is more common than infiltration of the Waldeyer ring or other extranodal presentation [11]. Four variants of FL are distinguished in the current WHO classification of 2017, namely in situ follicular neoplasia (presenting at any site), duodenal type FL, testicular FL, and diffuse FL (showing a predominantly diffuse growth pattern and lacking the distinguishing *IGH/BCL2* chromosomal translocation). In addition, primary cutaneous follicle center lymphoma (formerly called Crosti lymphoma) is recognized as a distinct entity, which usually affects skin of the head or trunk [7]. FL manifesting as primary splenic lymphoma (PSL) is very rare, and therefore its clinicopathologic features are still not well established. Since several reviews are published every year on FL, this article will focus on indolent primary FL of the spleen (subsequently abbreviated as PSFL), an uncommon and understudied lymphoproliferative disorder.

A literature review highlighting unique characteristics and therapeutic management of PSFL will be presented in the following sections.

## 2. Epidemiology

PSL comprise approximately 1% of all malignant neoplasms [12,13], when the disease is restricted to involvement of the spleen and hilar lymph nodes. However, a broader definition also encompassing liver, bone marrow or peripheral blood involvement, but lacking prominent distant adenopathy, is accepted as well.

Epidemiologically, one study showed the prevalence of PSL to be approximately 62%, where DLBCL was the most common lymphoid neoplasm (25%) followed by splenic MZL (15%) and FL (6%) [14]. Low-grade CD10-positive lymphoproliferative disorders encountered in splenic specimens belong most frequently to the category of FL (either primary or secondary). However, a subset of FL in the spleen represents aggressive disease (equivalent to DLBCL), considering that extranodal FL is most commonly a high-grade lymphoma that may lack expression (or translocation) of BCL2 [15,16]. However, duodenal-type FL represents an exception to this rule, due to an indolent course and constant expression of BCL2 [9].

The incidence of PSFL is unknown and may be overestimated because the vast majority of FL in the spleen represent secondary disease. In general, PSFL accounts for a small fraction of PSL and only a few case series are available on this entity [17,18,19].

In general, FL has an incidence of 2.1 per in the United States (SEER Follicular Lymphoma—Cancer Stat Facts (https://seer.cancer.gov/statfacts/html/follicular.html, accessed 16 October 2021), and is less common in eastern Europe, Asia and developing countries [7]. FL is 2–3 times more common in white than in black patients [9,20], and disproportionately affects females. The mean age at diagnosis (including all histologic grades) has been estimated to be 68 years, which is similar to nodal FL [17,18,21,22,23]. PSFL is rarely seen in patients younger than 50 years based on case series [17,18,22].

## 3. Clinical Presentation

Due to the rarity of PSFL, clinical features are incompletely defined. In contrast to nodal FL, most patients present with stage I/II disease [23]. A minority of cases show bone marrow, peripheral blood or liver infiltration [18]. In PSFL, isolated splenomegaly either asymptomatic or accompanied by upper abdominal discomfort is the rule [18]. B symptoms occur in a minority (less than 20%) of cases. Cytopenias (anemia and/or thrombocytopenia) secondary to hypersplenism or rarely due to bone marrow infiltration have been reported [18,22]. In contrast, nodal FL, which may involve the spleen, is usually accompanied by distant lymphadenopathy and more frequent systemic symptoms. The real frequency of PSFL remains to be established. As such, the disease may be incidentally found during work up for other medical conditions [24]. However, pathologic splenic rupture due to massive splenomegaly has been described [25]. Atypical lymphocytosis observed in peripheral blood smears or liver biopsies and/or mild to moderate splenomegaly may be identified incidentally during clinical encounters unrelated to lymphoma work-up in asymptomatic or paucisymptomatic patients. Interestingly, anti-hepatitis C virus (HCV) antibodies were more prevalent in patients with splenic FL compared with nodal FL with splenic infiltration in a study of 17 cases [18].

## 4. Morphologic Findings

**Figure 1:**
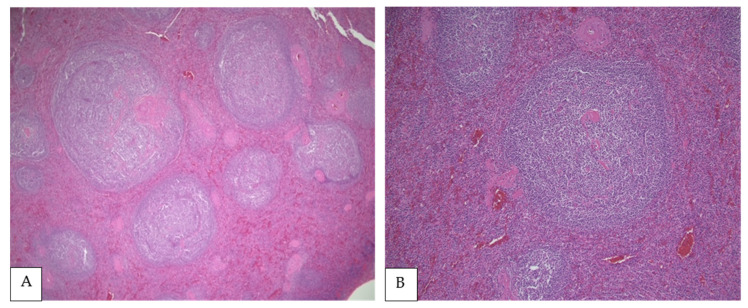

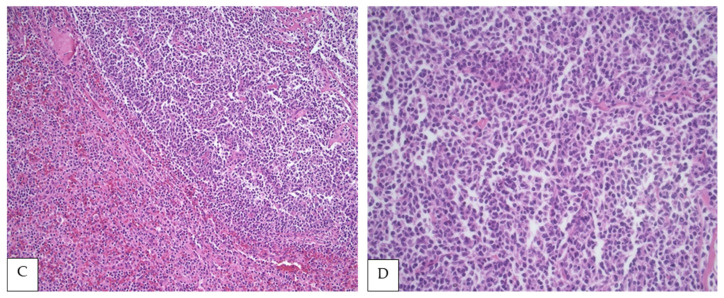
(**A**,**B**) PSFL in the white pulp of the spleen (H&E, 40× and 100×, respectively). (**C**) Interphase between nodular white pulp with lymphomatous involvement and red pulp without lymphoma (H&E, 200×), (**D**) Lymphoma cells composed mainly of centrocytes (H&E, 400×).

FL is morphologically composed of a heterogeneous lymphoid proliferation including small/medium sized cleaved cells with inconspicuous nucleoli (centrocytes) and larger cells with round/oval/convoluted vesicular nuclei, membrane bound nucleoli and visible cytoplasm (centroblasts) admixed in various proportions (Figure 1). Current grading (based on the average frequency of centroblasts from at least 10 high power fields) includes two tiers: low-grade/grade 1–2 FL (with less than 16 centroblasts) and high-grade/grade 3 FL (with 16 or more centroblasts). High-grade cases without centrocytes (grade 3B) tend to behave more aggressively paralleling DLBCL.

Due to a dearth of studies, it is uncertain if PSFL has unique morphologic features. Mollejo et al. analyzed 32 cases of FL in the spleen including an undetermined number of PSFL [22]. However, most cases (18) represented low-grade disease, and the cohort was divided according to expression of BCL2 (see the Immunophenotypic Findings). Regardless of grading, all cases showed a white pulp predominant micronodular pattern, but some cases also showed red pulp extension [22]. Howard et al. examined 16 cases of splenic lymphoma in which up to 10 cases represented PFSL, identifying two histological patterns irrespectively of the origin of the lymphoma (primary versus secondary) [15]. The first pattern demonstrated distortion of the normal splenic architecture with packed neoplastic follicles/nodules and some interfollicular proliferation. The second pattern showed preserved splenic architecture with subtle white pulp expansion and an intact red pulp. Interestingly, all six cases with intact architecture represented low-grade disease, which could have been misinterpreted as reactive follicular hyperplasia [17]. Lastly, Shimono et al. studied 17 patients with PSFL and 153 controls with nodal FL. Splenic macronodular lesions (larger than 3 cm) were less frequent in patients with nodal FL; however, architectural differences or histologic grade stratification were not highlighted in this report [18].

## 5. Immunophenotypic Findings

**Figure 2:**
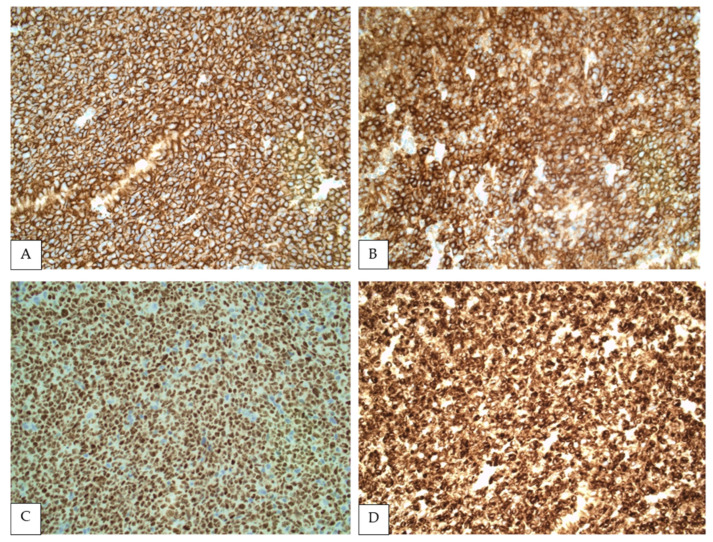
Immunohistochemistry showing FL cells strongly positive for CD20 (**A**), CD10 (**B**), BCL6 (**C**) and BCL2 (**D**) (All at 400×).

Like other mature B cell neoplasms, FL expresses pan-B cell antigens (CD19, CD22, CD79a and PAX5), and is also by definition positive for germinal center markers, such as BCL6, HGAL and LMO2. Accordingly, splenic FL almost always co-expresses CD20 and BCL6 (Figure 2) [17,22]. However, BCL6 negativity may be observed in high-grade FL [26], and this marker is frequently downregulated in the spleen [27].

In addition, BCL2 co-expression is variable. It is present in the vast majority of low-grade cases but may be absent in higher grade FL [22]. Similarly, BCL2 expression fluctuates depending on the topography of FL [9,28], which reflects on splenic FL. In a study of 32 cases of splenic FL, BCL2 was reported positive in 37.5%, mostly representing PSFL [22]. Other studies demonstrated BCL2 expression in 82.4% [18] and 100% of PSFL [17]. Notably, BCL2 expression may correlate with prognosis, since negative cases were enriched for higher grade morphology (including five cases transformed to DLBCL) when compared with BCL2-positive cases [22]. CD10 is also expressed in most PSFL ranging from 58.1% [22] to 100% of cases in published series [18]. MUM-1 expression was detected only in 0% to 13% of cases [18,22]. Newer germinal center B cell markers have been developed, including HGAL and LMO2, which are not site specific. In a study with 13 cases of FL in the spleen, these markers were reported to be less sensitive than CD10, limiting their diagnostic utility in splenic tissue [29]. CD43 is generally negative in FL, and therefore immunoreactivity for this marker in the absence of expression of germinal center antigens may preclude the diagnosis of PSFL [30].

Finally, increased seropositivity for HCV has been detected in many types of mature B cell lymphoma, including FL, but a definite viral pathogenic role has not been established in this lymphoma [31]. A study of 17 patients reported a significant association with HCV infection in PSFL versus nodal FL, but immunohistochemistry was not performed to detect viral protein expression in tumor cells, and an etiopathogenic relationship was not further investigated [18]

Emerging and historic data corroborates that incorporation of Ki-67 proliferation index serves as an adjunct prognostic indicator in FL by facilitating centroblast identification, and by acting as an independent progression free survival (PFS) marker uncoupled from histologic grading [32,33,34].

## 6. Genetic Findings

Limited studies have been performed on genetic profiling of PSFL, and a systematic comparison with nodal FL has not been published. Various chromosomal alterations (most commonly loss of 1p, 6p, 10q and 17p; or gains of 1, 6p, 7, 8, 12q, X and 18q) are seen in FL [9]. Two independent studies reported a high degree of overlap between nodal and splenic FL regarding pathological and cytogenetic features [17,18]. The characteristic t(14;18) translocation juxtaposing *IGH* and *BCL2* is present in approximately 90% of low-grade FL [9], and shows a good correlation with BCL2 expression by immunohistochemistry [35].

Interestingly, Mollejo et al. reported that 50% of PSFL expressing BCL2 also exhibit the *IGH/BCL2* translocation and represent predominantly low-grade histology [22]. Of note, BCL6 rearrangements, which can be present in DLBCL, were absent in PSFL [22]. Another study using conventional fluorescent in situ hybridization, detected *IGH/BCL2* translocations in 91% of 11 cases, and a complex cryptic 3-way translocation involving *BCL6*, *IGH* and 11q (excluding *CCND1*) in one additional case [17]. Shimono et al. also found genetic similarity (based in *IGH-BCL2*, *NOTCH1* and *NOTCH2* analysis) between nodal and splenic lymphoma in a series studying 17 patients with PSFL [18]. Splenomegaly, female sex, and absence of *IGH/BCL2* translocation are associated with higher frequency of *NOTCH* mutations in FL [36]. However, *NOTCH1/NOTCH2* alterations were detected only in 6.3% of 11 cases in a small study [36]. Therefore, additional research is necessary to determine if splenic FL has a unique molecular landscape selectively affecting genes commonly mutated in FL, such as: *BCL2*, *KMT2D (MLL2)*, *TNFRSF14*, *EZH2*, *EPHA7*, *CREBBP*, *BCL6* and *TNFAIP3* [9].

## 7. Diagnostic Work-Up

Comprehensive clinical and laboratory assessment (including viral hepatitis and human immunodeficiency virus tests), imaging studies (ideally CT and PET/CT) and bone marrow/peripheral blood evaluation (including multiparametric immunophenotyping) are strongly recommended if splenic FL is suspected during the clinical investigation of isolated splenomegaly [37]. Ultrasound imaging has also been used in the diagnostic workup with an overall sensitivity of approximately 50% [38]. Flow cytometry is extremely useful in the setting of PSFL due its high sensitivity to detect minimal involvement of peripheral blood and/or bone marrow. Similarly, ancillary cytogenetic and molecular diagnostic techniques may be invaluable to identify the t(14;18) translocation and other genetic alterations. Ultimately, histologic examination of spleen may be necessary, since FL is usually cytologically heterogeneous, and sampling errors due to inadequate tissue (particularly from core biopsies) may result in misdiagnosis. For instance, a significant number of DLBCL may selectively involve the bone marrow with clones composed only by small cells, mimicking low-grade lymphoma [39]. Similarly, splenic core biopsies may incorrectly suggest the diagnosis of PSFL (or DLBCL) due to sampling bias in heterogeneous tumors. Therefore, splenectomy may eventually be necessary for a precise diagnosis of lymphoma, including PSFL.

## 8. Differential Diagnosis

The combination of morphology and immunophenotype generally yields a categorical diagnosis and subtyping of B cell lymphoma. The differential diagnosis of FL is narrow after excluding high-grade CD10-positive lymphomas, such as DLBCL and BL, which are beyond the scope of this review. Clinicopathological correlation is necessary to determine if FL represents primary or secondary disease. Morphologically, PSFL is most frequently composed by small cells (centrocytes) without sheets of larger cells. In contrast, DLBCL and BL show sheets of large- and medium-sized cells, respectively. Furthermore, while PSFL, BL and DLBCL predominantly involve white pulp, DLBCL may spill into red pulp [40]. Interestingly, BL presenting as PSL would be very unusual [41].

Excellent reviews are available to guide a practical immunophenotypic subclassification of B cell lymphoma [42,43], which are applicable to PSFL. Interestingly, a hierarchical model based on binary expression of CD5 and CD10 has also been proposed using machine learning technology [44]. In summary, FL is usually not a diagnostic dilemma by virtue of observing a distinctive immunophenotype (positivity for CD10, BCL2, BCL6, HGAL, LMO2, GCET1 and Stathmin, and negativity for CD5, CD43 and CyclinD1) in the correct morphologic setting (see the Morphologic Findings). However, if a rare CD10-negative FL is considered in the differential diagnosis, additional markers such as IRTA and MNDA (which are commonly positive in MZL and negative in FL) may be necessary [45]. Other CD5/CD10-double negative low-grade B cell lymphomas may very infrequently show aberrant expression of CD10, posing a diagnostic challenge to differentiate from FL (particularly with splenic presentation), as covered extensively elsewhere and in this review series [42,43,44,45,46] (and cross referencing Auerbach, Gomes and Aggarwal’s papers). For example, HCL can express CD10 [7], but its triple positivity for CD25, CD103 and CD123 together with the absence of BCL6 expression (usually positive in FL), and the presence of *BRAF* V600E alteration, should permit clear segregation from other small B cell lymphomas, including PSFL [7]. Similar diagnostic capacity can be attributed to the MYD88 L265P mutation in combination with an IgM gammopathy to correctly identify LPL [47].

## 9. Treatment and Prognosis

Treatment of splenic lymphomas is ascribed to its lineage. Based on a few available studies it has been suggested to treat patients with splenic lymphomas with regimens developed for nodal lymphomas, which is particularly true for FL. However, long term outcome data are unavailable given the rarity of splenic lymphomas. Consequently, specific treatment modalities are not formally recommended for PSFL yet [37].

HCV infection has been more frequently described in PSFL than in nodal FL [18], and anti-viral therapy alone can induce remission of lymphoproliferative disorders [48,49,50], suggesting this option for PSFL.

Stage I PSFL diagnosed on splenectomy specimen could be subjected to an active observation/expectant management strategy, mirroring guidelines for nodal FL stage I treated with complete surgical removal of the affected lymph nodes [37]. Long treatment-free intervals may be achieved by this approach, while minimizing therapy-related adverse effects. A recent large case series of splenic lymphomas undergoing splenectomy demonstrated that additional chemotherapy after surgery did not impact overall survival (OS) in FL [23], further supporting the practice of expectant management. However, the management of stage I nodal FL remains mired in controversy. While current guidelines still recommend localized radiotherapy, heterogenous alternatives (ranging from vigilance to adjuvant Rituximab monotherapy or immunochemotherapy) are used as evident from prospective observational studies and real-world series [51]. The combination of radiotherapy with systemic treatments (chemotherapy or immunochemotherapy) has been shown to prolong PFS but not OS, and the decision to offer additional treatment remains both patient and physician dependent [52,53]. These concepts have been applied to early PSFL. On the other hand, PSFL presenting with combinations of liver, bone marrow and peripheral blood involvement are expected to behave like advanced FL (stage IV), and the therapeutic choices range from active observation (for low tumor burden asymptomatic disease) to first line immunochemotherapy (for high tumor burden symptomatic disease) [37]. Commonly, combinations of Rituximab with Bendamustine, CHOP or CVP have been used as first line regimens [54,55]. Recently, substituting Rituximab for Obinutuzumab (a type II glycoengineered humanized anti-CD20 monoclonal antibody) in similar chemotherapy combinations was shown to prolong PFS without impacting OS [56].

Maintenance regimens with anti-CD20 monoclonal antibodies have been applied by several investigators, again prolonging PFS but not OS [57]. The efficacy of maintenance regimens for advanced leukemic disease (as is common in stage IV PSFL) deserves investigation, since lymphocytosis at presentation implies poor prognosis in nodal FL [58,59]. Treatment of relapsed PSFL can also be modelled on nodal FL, where management is influenced by multiple, patient and disease related factors, including the interval between diagnosis and relapse, and documentation of histologic transformation. A growing number of signaling pathway-specific/non-chemotherapy agents and cellular therapies are becoming available for FL, which could change the course of this still incurable disease [60,61]. However, the applicability of these new strategies to PSFL is unknown, and probably dependent on further clarification of specific biological characteristics and pathogenic mechanisms.

Conclusive data on prognostication of PSFL are not available yet. In the largest series published to date, survival was similar to nodal FL using treatment options ranging splenectomy alone to combined surgery and chemotherapy [22]. It is conceivable that the prognosis of PSFL may be influenced by the stage of disease and prognostic scores usually applied to nodal FL (namely the Follicular Lymphoma International Prognostic Index/FLIPI and FLIP2) [62,63]. However, the clinical outcomes of patients with spleen-confined disease have been similar to those of individuals with disseminated lymphoma [17], which may be explained by a limited sample size.

When PFSL transforms to aggressive lymphoma, regimens for DLBCL (immunochemotherapy with anti-CD20 antibodies and standard anthracycline-based chemotherapy for at least four cycles in localized stages) are used achieving comparable results.

## 10. Conclusions

In conclusion, novel chemotherapy-free modalities emerging for other types of B cell lymphoma will be adapted for splenic lymphomas, enabling improvements in the treatment and prognosis of PSFL that may evolve in parallel with a more detailed biological characterization of this rare entity.
